# Acute Exercise Increases Adiponectin Levels in Abdominally Obese Men

**DOI:** 10.1155/2012/148729

**Published:** 2012-05-30

**Authors:** Travis J. Saunders, Andrew Palombella, K. Ashlee McGuire, Peter M. Janiszewski, Jean-Pierre Després, Robert Ross

**Affiliations:** ^1^Healthy Active Living and Obesity Research, Children's Hospital of Eastern ON Research Institute, 401 Smyth Road, Ottawa, ON, Canada K1H 8L1; ^2^School of Human Kinetics, Faculty of Health Sciences, University of Ottawa, Ottawa, ON, Canada K1N 6N5; ^3^Department of Anatomy & Cell Biology, The University of Western Ontario, London, ON, Canada N6A 5C1; ^4^School of Kinesiology and Health Studies, Queen's University, Kingston, ON, Canada K7L 3N6; ^5^Centre de Recherche de l'Institut Universitaire de Cardiologie et de Pneumologie de Québec, 2725, Chemin Sainte-Foy, Québec, QC, Canada G1V 4G5; ^6^Division of Kinesiology, Department of Social and Preventive Medicine, Laval University, Québec, QC, Canada G1V0 A6; ^7^Division of Endocrinology and Metabolism, Department of Medicine, Queen's University, Kingston, ON, Canada K7L 3N6

## Abstract

*Objective*. To examine the effect of acute and short-term (~1 week) aerobic exercise training on plasma adiponectin levels in inactive, abdominally obese men. *Materials and Methods*. Inactive and abdominally obese men (*n* = 38, waist circumference ≥102 cm) recruited from Kingston, Canada were randomly allocated to perform three bouts of aerobic treadmill exercise at either low (50% VO_2_ peak) or high (75% VO_2_ peak) intensity during a 1-week period. Blood samples were taken before and after the first exercise session and 24–72 hours following the completion of the final exercise session. *Results*. Adiponectin levels were elevated immediately following an acute bout of exercise at both high and low intensities (High: 5.79 ± 0.42 versus 5.05 ± 0.41 ug/mL; Low: 5.24 ± 0.44 versus 4.37 ± 0.44 ug/mL, *P* < 0.05) and remained elevated following 30 minutes of rest. In comparison to baseline, adiponectin levels were also elevated 24–72 hours following the final exercise session (High: 5.47 ± 0.48 versus 4.88 ± 0.48 ug/mL; Low: 5.18 ± 0.49 versus 4.47 ± 0.49 ug/mL, *P* < 0.05). *Conclusion*. Both acute and short-term aerobic exercise result in a significant increase in plasma adiponectin levels in inactive, abdominally obese men independent of intensity.

## 1. Introduction

Obesity and related metabolic disorders are among the most important public-health concerns in developed nations [1]. One possible target for the reduction of obesity-related cardiometabolic risk is adiponectin, an adipokine with insulin-sensitizing and antiatherogenic properties, which circulates at levels inversely proportional to both total and abdominal fat mass [2–4]. Evidence suggests that an acute bout of vigorous aerobic exercise may result in a significant increase in plasma adiponectin levels in trained athletes, although this increase is not apparent until 30 minutes after the cessation of exercise [5–7]. In contrast, immediately following the cessation of exercise, adiponectin levels are reported to be unchanged [5] or even reduced [7] in trained individuals. Further, evidence also suggests that an acute bout of aerobic exercise at a moderate intensity has little or no impact on adiponectin levels in healthy, but untrained populations [8].

At present the relationship between an acute bout of aerobic exercise and adiponectin levels in inactive, abdominally obese individuals is unclear. Two studies have examined adiponectin levels immediately after exercise in this population [9, 10] and report that adiponectin levels are decreased [10] or unchanged [9]. However, to date no studies have examined adiponectin levels in abdominally obese individuals 30 minutes after the cessation of an acute bout of aerobic exercise, the time point when adiponectin has been reported to be increased in athletic populations [5, 7].

At present it is also unclear whether one week of aerobic exercise training is associated with an increase in plasma adiponectin levels in individuals with abdominal obesity. Kriketos et al. [11] report that 2 to 3 bouts of aerobic exercise spread over one week resulted in a dramatic 11.2 *μ*g/mL (equivalent to 160% of baseline values) increase in plasma adiponectin levels in inactive, abdominally obese men. An increase of this magnitude, however, is difficult to reconcile with other evidence. A recent systematic review reports that the average adiponectin increase observed in response to chronic exercise across all populations is just 1.33 *μ*g/mL, with the majority of studies, including those involving up to 24 weeks of regular exercise, failing to observe a significant change in adiponectin levels following the intervention [8]. Further, investigations of pharmacotherapy suggest similar small-to-moderate effects on adiponectin levels [12]. To our knowledge, the effect of a single week of aerobic training on adiponectin levels in a population of inactive and abdominally obese individuals has yet to be investigated, or corroborated, by any subsequent interventions.

Given the high prevalence of both hypoadiponectinemia and cardiometabolic risks among abdominally obese individuals [4], it is important to improve our understanding of the relationship between exercise and adiponectin levels in this high-risk population. The purpose of the present study was to examine the effect of both acute and short-term (~1 week) aerobic exercise training on plasma adiponectin levels in inactive, abdominally obese men. We also sought to determine whether exercise intensity influences the response to plasma adiponectin levels in this population, independent of caloric expenditure. We hypothesized that adiponectin levels would be significantly decreased immediately following a bout of acute exercise, but significantly increased 30 minutes after the cessation of exercise, as well as after one week of aerobic training. We also hypothesized that for a given energy expenditure, exercising at 75% of VO_2_ peak would have a greater impact on adiponectin levels than exercising at 50% of VO_2_ peak.

## 2. Materials and Methods

### 2.1. Participants

Participants included 38 abdominally obese men (waist circumference ≥102 cm) aged 25 to 50 years. All participants reported being weight stable (±1 kg) for 6 months prior to the study and engaged in physical activity less than once per week. Potential participants were excluded from the study if they reported smoking or had a history of heart disease, stroke, or diabetes or were taking glucose-lowering medication. The use of lipid-lowering (e.g., statins) and hypertension (e.g., angiotensin-converting enzyme inhibitors) medications was permitted. All participants gave their informed written consent before participation in accordance with the ethical guidelines set by Queen's University. Participants were instructed to maintain their usual diet throughout the duration of the study.

### 2.2. Anthropometric Measurements

Body mass was measured to the nearest 0.1 kg on a calibrated balance. Standing height was measured to the nearest 0.1 cm with the use of a wall-mounted stadiometer. Waist circumference (WC) was measured at the superior border of the iliac crest and was taken to the nearest 0.1 cm after a normal expiration.

### 2.3. Cardiorespiratory Fitness

Cardiorespiratory fitness (measured as oxygen consumption per unit of time (VO_2_ peak)) was determined using results of a maximal treadmill test combined with standard open-circuit spirometry techniques (SensorMedics Corp, Yorba Linda, California) at baseline. Fitness testing took place at least one week prior to the beginning of the exercise protocol for all participants.

### 2.4. Exercise Protocol

Participants were randomly allocated to High- or Low-intensity treadmill exercise groups using a random number table. Group allocation was not revealed to individual participants until they arrived at their first exercise session. The High-intensity group exercised at a heart rate approximating an intensity of 75% of VO_2_ peak, while the Low-intensity group exercised at an intensity of 50% VO_2_ peak, as determined by the prestudy fitness test. The length of the exercise session was adjusted to ensure that each participant expended an estimated 400 calories per exercise session in order to investigate the impact of exercise intensity while keeping expenditure constant. Participants in both groups performed three bouts of aerobic exercise within one week, with one- to two-days of rest between each session.

### 2.5. Blood Samples and Biochemistry

All blood samples were taken following an overnight 12-hour fast. 10 mL of blood was taken from the antecubital vein immediately before, immediately after, and 30 minutes after the completion of the first exercise session. Blood samples were also taken 24–72 hours following the third and final exercise session. Plasma glucose was measured using an automated glucose analyzer (YSI 2300 Glucose Analyzer; YSI, Yellow Springs, OH). HDL-cholesterol and triglyceride levels were measured using enzymatic methods on the Roche Modular analytical system (Roche Diagnostics, Indianapolis, IN). Plasma adiponectin concentrations were determined using an enzyme-linked immunosorbent assay (B-Bridge International, Inc., San Jose, CA) of whole plasma kept at −80°C before use. All adiponectin measurements were performed centrally at the Cardiovascular Risk Factor Laboratory at the Quebec Heart and Lung Institute at Université Laval, Québec, Canada, and samples were shipped frozen using dry ice. Shifts in plasma volume during the acute bout of exercise were calculated using the method outlined by Dill and Costill [13].

### 2.6. Statistics

*T*-tests were used to compare baseline values between the two groups. A mixed-effect model with random intercept was used to assess changes in markers of cardiometabolic risk following a single session of aerobic exercise, as well as 24–72 hours after the third and final session of aerobic exercise. The following variables were included in the mixed-effect model for acute exercise: time of blood draw, group of participant, and changes in plasma volume. For short-term exercise, changes in plasma volume were omitted from the model. Statistical significance was defined as a *P* value of 0.05 or less, and a Bonferroni correction was used to adjust for multiple comparisons in post hoc tests following the mixed-effect model. All analyses of acute changes are controlled for changes in plasma volume.

 Variables which exhibited nonnormal distributions were transformed prior to analysis using a log transformation. Five participants (3 from the High group, 2 from the Low group) participated in only the first exercise session and were thus excluded from analyses examining the impact of 3 exercise sessions on markers of metabolic risk. All analyses were performed using SAS 9.2 and SPSS 18.0.

## 3. Results

 Participant characteristics are presented in Table 1.  Table 2 displays values of adiponectin and other markers of cardiometabolic risk before and after a single session of aerobic exercise. Exercise at either a High- or Low-intensity resulted in a significant increase in adiponectin levels both immediately (High: *P* = 0.003; Low: *P* < 0.0001) and 30 minutes after exercise (High: *P* = 0.0137; Low: *P* = 0.008). There was no significant group by time interaction (*P* = 0.8276). In comparison to baseline, triglyceride levels remained unchanged immediately following exercise in both groups (High: *P* = 0.2103; Low: *P* = 1.0) but were significantly decreased in the High-intensity group 30 minutes subsequent to exercise (*P* = 0.009). There was a borderline significant reduction of insulin levels immediately following the cessation of exercise in the Low-intensity group (*P* = 0.053), although they were no different from baseline 30 minutes following the cessation of exercise (*P* = 1.0). No changes in insulin levels were observed in the High-intensity group (*P* > 0.05). Acute exercise did not result in any significant changes in any other marker of cardiometabolic risk (see Table 2), and changes in adiponectin were not associated with changes in any other marker of cardiometabolic risk (data not shown, *P* > 0.05).

**Figure 1:**
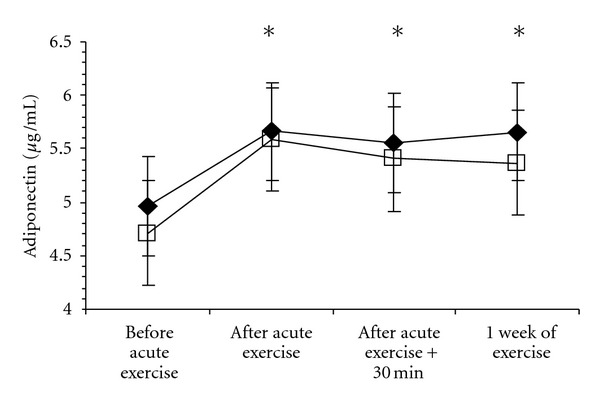
Changes in adiponectin following aerobic exercise in abdominally obese men. *Significantly different than before exercise in both groups, *P* < 0.05. Groups are presented as High- (*◆*) and Low- (□) intensity exercise. Plasma volume was not assessed at the final blood draw, and thus values in this figure have not been adjusted for changes in plasma volume.

Table 3 presents the levels of adiponectin and other markers of cardiometabolic risk before and after three sessions of aerobic exercise training at either a High or Low intensity. As expected, there were no significant changes in body weight or waist circumference in either group following one week of aerobic training (*P* > 0.05). Adiponectin levels were significantly elevated in all participants 24–72 hours after the final exercise session (*P* < 0.05), and these values were not significantly different from those observed following an acute bout of aerobic exercise (*P* > 0.05) (see Figure 1). There was no significant group by time interaction (*P* = 0.6635). Three sessions of aerobic exercise did not result in significant changes in any other marker of cardiometabolic risk (*P* > 0.05). Changes in adiponectin were not associated with changes in any marker of cardiometabolic risk except for fasting glucose (*r* = 0.40, *P* = 0.0234).

## 4. Discussion

 The present findings support our original hypotheses and suggest that an acute bout of aerobic exercise results in a significant increase in plasma adiponectin levels in abdominally obese men. Further, three sessions of aerobic exercise within a one-week period were sufficient to maintain this increase for 1–3 days after the final exercise session. It is worth noting that the increases in adiponectin observed in the present study occurred in the absence of any changes in weight or waist circumference. As such, these findings add to the growing body of evidence showing that exercise results in important health benefits irrespective of changes in body weight [14, 15].

The current findings are in agreement with previous work which has reported that vigorous exercise is associated with an acute increase in plasma adiponectin levels in trained athletes [5, 7]. These results also support the findings of Kriketos et al. [11], who report that one week of aerobic training results in increased adiponectin levels in abdominally obese men. It should be noted, however, that the increase in adiponectin levels following one week of training in the present study was of a much smaller magnitude than what has been reported previously [11].

 In contrast to our hypothesis, the increase in adiponectin levels following a session of acute exercise was detectable immediately following the cessation of exercise. These results differ from the recent findings of Numao and colleagues [10], who report that adiponectin levels in abdominally obese men are significantly reduced immediately following the cessation of aerobic exercise at a high intensity and unchanged immediately following aerobic exercise at a low intensity. The reasons for these discrepant findings are not immediately clear. One difference worth noting, however, was that participants in the present study were almost exclusively Caucasian, while those involved in the study of Numao and colleagues were Japanese. Adiponectin has been shown to circulate in isoforms of varying molecular weights [16], and it has been reported that the relative proportions of these isoforms, and their relationships with disease, differ by ethnicity [17, 18]. For example, it has been reported that Indo-Asian women have a significantly lower proportion of high molecular weight adiponectin than their Caucasian peers [18]. Of note, Numao and colleagues report that although circulating levels of both medium- and low-molecular weight adiponectin decreased immediately following a bout of vigorous aerobic exercise, the proportion of high molecular weight adiponectin was significantly increased [10]. Thus, it is possible that ethnic differences in the relative proportions of adiponectin isoforms, or the response of these isoforms to acute exercise, may influence the relationship between exercise and total adiponectin levels. Unfortunately, the distribution of adiponectin isoforms was not assessed in the present study.

 Differences between the current findings and those of Numao may also be related to the mode of exercise employed by the two studies. To our knowledge, only studies which have employed vigorous whole-body exercise report an acute increase in plasma adiponectin levels [5, 7]. In contrast, participants in the recent report by Numao et al. [10] performed exercise on a cycle ergometer, which may have engaged fewer muscle groups than the current intervention. Future research is needed to clarify the reasons behind these divergent findings.

### 4.1. Potential Mechanisms

 Although it was once thought that adiponectin was produced exclusively in adipocytes, recent evidence suggests that adiponectin is also actively secreted by myocytes [19–21]. Further, Amin and colleagues have shown that the PPAR*γ* agonist rosiglitazone induces a significant increase in muscle adiponectin production [19]. Acute exercise has also been shown to result in rapid changes in the transcription of PPAR*γ* coactivator Pgc-1*α* in skeletal muscle, suggesting that this pathway may link acute exercise with adiponectin production at the level of the myocyte [22, 23]. However, to our knowledge no studies have yet examined the role of exercise in adiponectin production in muscle cells.

 Although both insulin and catecholamine levels have been suggested to play a role in acute changes in adiponectin levels [7, 10, 24], they seem unlikely to explain the increases in adiponectin observed in the present study. In the present study, adiponectin levels were elevated above baseline in both groups at all postexercise time points, while insulin levels were only reduced at one time point (immediately following exercise) in one group (Low). This suggests that changes in insulin levels are not the primary mediator of the observed change in adiponectin levels. The role of catecholamines in this process is also unclear. While they are thought to play a largely inhibitory role in adiponectin expression [10, 25], it has also been shown that catecholamine levels are *increased* following vigorous exercise [26], which suggests that they too are unlikely to explain the present findings.

 The present study has several strengths and limitations which warrant mention. As mentioned previously, the present analysis focused solely on total adiponectin concentrations, rather than looking at concentrations of specific adiponectin oligomers. The current study also focused exclusively on a relatively homogeneous group of inactive, abdominally obese adult males, and thus our findings may not translate to females or to individuals with ages or phenotypes different from those of participants in the current sample. While this may limit the generalizability of these results to other groups, the population studied in the current investigation is at substantially increased risk of hypoadiponectinemia and cardiometabolic dysfunction [4]. As a result, interventions which are able to increase adiponectin levels in this population could have important clinical applications. Finally, the current investigation also benefits from a randomized design and the tight monitoring and control of exercise intensity for participants in both groups.

 In summary these findings suggest that an acute bout of exercise, regardless of exercise intensity, results in a significant increase in plasma adiponectin levels in inactive, abdominally obese men. Further, an acute bout of exercise at high, but not low, intensity also results in a significant reduction in plasma triglyceride levels in these same individuals. Finally, adiponectin levels remained elevated 24–72 hours after completing 3 exercise sessions in a one-week period. These changes were observed in the absence of any change in weight or body composition. These results suggest that positive changes in adiponectin levels may be yet another important health benefit observed with even a single session of aerobic exercise.

## Figures and Tables

**Table 1:** Participant characteristics.

	Low (*n* = 18)	High (*n* = 20)
Age	38.39 (11.2)	38.95 (7.6)
BMI (kg/m^2^)	33.00 (3.8)	33.96 (3.6)
Waist circumference (cm)	112.97 (8.7)	117.10 (8.9)
Systolic blood pressure (mmHg)	124.33 (15.7)	116.65 (30.1)
Diastolic blood pressure (mmHg)	82.94 (7.8)	77.2 (19.8)
VO_2_ peak (mL/kg/min)	32.00 (4.59)	32.83 (6.1)
Exercise volume (min)	48.6 (6.6)	30.75 (6.1)*
Average intensity (% of max)	47.9% (3.9)	72.6% (4.3)*
Calories burned (kcal)	383.32 (36.2)	394.4 (29.7)

Data are presented as mean (Standard Deviation).

*Significantly different from the Low group, *P* ≤ 0.05.

**Table 2:** Acute changes in markers of cardiometabolic risk following one bout of aerobic exercise.

	Group	Before exercise	Following exercise	Following exercise + 30
Adiponectin (ug/mL)	High	5.05 (0.41)	5.79 (0.42)*	5.69 (0.42)*
	Low	4.37 (0.44)	5.24 (0.44)*	5.11 (0.44)*
Triglycerides (mmol/L)	High	1.98 (0.24)	2.10 (0.24)	1.77 (0.24)*
	Low	2.04 (0.26)	2.09 (0.26)	1.93 (0.26)
HDL cholesterol (mmol/L)	High	1.04 (0.053)	1.06 (0.054)	0.99 (0.053)
	Low	0.81 (0.056)	0.88 (0.056)	0.84 (0.059)
LDL cholesterol (mmol/L	High	3.62 (0.23)	3.60 (0.23)	3.53 (0.23)
	Low	2.89 (0.25)	2.90 (0.25)	2.86 (0.25)
Insulin (pmol/L)	High	85.05 (7.74)	85.63 (7.47)	91.63 (9.11)
	Low	108.38 (24.67)	68.25 (11.27)	79.44 (14.85)
Glucose (mmol/L)	High	5.44 (0.15)	5.60 (0.16)	5.45 (0.15)
	Low	4.83 (0.17)	4.75 (0.17)^†^	4.73 (0.17)^†^

Data presented as mean (standard error). All acute data are controlled for changes in plasma volume.

*Significantly different from baseline, *P* ≤ 0.05, using Bonferroni correction.

^†^Significantly different from High-intensity group at the same time point *P* ≤ 0.05, using Bonferroni correction.

**Table 3:** Changes in markers of cardiometabolic risk following one week of aerobic exercise training.

	Group	Before training	After training
Adiponectin (ug/mL)	High	4.86 (0.48)	5.47 (0.48)*
	Low	4.47 (0.49)	5.18 (0.49)*
Triglycerides (mmol/L)	High	2.07 (0.27)	1.71 (0.27)
	Low	2.06 (0.28)	2.27 (0.27)
HDL Cholesterol (mmol/L)	High	1.04 (0.059)	1.00 (0.06)
	Low	0.77 (0.061)^†^	0.95 (0.06
LDL Cholesterol (mmol/L	High	3.58 (0.25)	3.48 (0.25)
	Low	2.91 (0.25)	3.07 (0.25)
Insulin (pmol/L)	High	85.05 (7.74)	73.00 (10.52)
	Low	108.38 (24.67)	90.13 (15.15)
Glucose (mmol/L)	High	5.36 (0.15)	5.55 (0.15)
	Low	4.92 (0.17)	5.05 (0.16)
Weight (kg)	High	114.48 (4.03)	114.49 (4.03)
	Low	105.42 (4.16)	104.72 (4.16)
Waist Circumference (cm)	High	118.50 (2.07)	117.55 (2.07)
	Low	112.92 (2.13)	112.71 (2.14)

Data presented as mean (standard error).

*Significantly different from baseline, *P* ≤ 0.05, using Bonferroni correction.

^†^Significantly different from High-intensity group at the same time point *P* ≤ 0.05, using Bonferroni correction.

## References

[1] Jia H, Lubetkin EI (2010). Trends in quality-adjusted life-years lost contributed by smoking and obesity. *American Journal of Preventive Medicine*.

[2] Saunders TJ, Davidson LE, Janiszewski PM, Després JP, Hudson R, Ross R (2009). Associations of the limb fat to trunk fat ratio with markers of cardiometabolic risk in elderly men and women. *Journals of Gerontology A*.

[3] Gavrila A, Chan JL, Yiannakouris N (2003). Serum adiponectin levels are inversely associated with overall and central fat distribution but are not directly regulated by acute fasting or leptin administration in humans: cross-sectional and interventional studies. *Journal of Clinical Endocrinology and Metabolism*.

[4] Després JP (2006). Is visceral obesity the cause of the metabolic syndrome?. *Annals of Medicine*.

[5] Jürimäe J, Hofmann P, Jürimäe T (2006). Plasma adiponectin response to sculling exercise at individual anaerobic threshold in college level male rowers. *International Journal of Sports Medicine*.

[6] Bouassida A, Chamari K, Zaouali M, Feki Y, Zbidi A, Tabka Z (2010). Review on leptin and adiponectin responses and adaptations to acute and chronic exercise. *British Journal of Sports Medicine*.

[7] Jürimäe J, Purge P, Jürimäe T (2005). Adiponectin is altered after maximal exercise in highly trained male rowers. *European Journal of Applied Physiology*.

[8] Simpson KA, Singh MAF (2008). Effects of exercise on adiponectin: a systematic review. *Obesity*.

[9] Jamurtas AZ, Theocharis V, Koukoulis G (2006). The effects of acute exercise on serum adiponectin and resistin levels and their relation to insulin sensitivity in overweight males. *European Journal of Applied Physiology*.

[10] Numao S, Katayama Y, Hayashi Y, Matsuo T, Tanaka K (2011). Influence of acute aerobic exercise on adiponectin oligomer concentrations in middle-aged abdominally obese men. *Metabolism*.

[11] Kriketos AD, Gan SK, Poynten AM, Furler SM, Chisholm DJ, Campbell LV (2004). Exercise increases adiponectin levels and insulin sensitivity in humans. *Diabetes Care*.

[12] Riera-Guardia N, Rothenbacher D (2008). The effect of thiazolidinediones on adiponectin serum level: a meta-analysis. *Diabetes, Obesity and Metabolism*.

[13] Dill DB, Costill DL (1974). Calculation of percentage changes in volumes of blood, plasma, and red cells in dehydration. *Journal of Applied Physiology*.

[14] Thompson PD, Crouse SF, Goodpaster B, Kelley D, Moyna N, Pescatello L (2001). The acute versus the chronic response to exercise. *Medicine and Science in Sports and Exercise*.

[15] Duncan GE, Perri MG, Theriaque DW, Hutson AD, Eckel RH, Stacpoole PW (2003). Exercise training, without weight loss, increases insulin sensitivity and postheparin plasma lipase activity in previously sedentary adults. *Diabetes Care*.

[16] Waki H, Yamauchi T, Kamon J (2003). Impaired multimerization of human adiponectin mutants associated with diabetes. Molecular structure and multimer formation of adiponectin. *Journal of Biological Chemistry*.

[17] Lara-Castro C, Doud EC, Tapia PC (2008). Adiponectin multimers and metabolic syndrome traits: relative adiponectin resistance in African Americans. *Obesity*.

[18] Retnakaran R, Hanley AJG, Connelly PW, Maguire G, Sermer M, Zinman B (2006). Low serum levels of high-molecular weight adiponectin in Indo-Asian women during pregnancy: evidence of ethnic variation in adiponectin isoform distribution. *Diabetes Care*.

[19] Amin RH, Mathews ST, Camp HS, Ding L, Leff T (2010). Selective activation of PPAR*γ* in skeletal muscle induces endogenous production of adiponectin and protects mice from diet-induced insulin resistance. *American Journal of Physiology*.

[20] Liu Y, Chewchuk S, Lavigne C (2009). Functional significance of skeletal muscle adiponectin production, changes in animal models of obesity and diabetes, and regulation by rosiglitazone treatment. *American Journal of Physiology*.

[21] Krause MP, Liu Y, Vu V (2008). Adiponectin is expressed by skeletal muscle fibers and influences muscle phenotype and function. *American Journal of Physiology*.

[22] Akimoto T, Pohnert SC, Li P (2005). Exercise stimulates Pgc-1*α* transcription in skeletal muscle through activation of the p38 MAPK pathway. *Journal of Biological Chemistry*.

[23] Pilegaard H, Saltin B, Neufer DP (2003). Exercise induces transient transcriptional activation of the PGC-1*α* gene in human skeletal muscle. *Journal of Physiology*.

[24] Imbeault P, Dépault I, Haman F (2009). Cold exposure increases adiponectin levels in men. *Metabolism*.

[25] Fasshauer M, Klein J, Neumann S, Eszlinger M, Paschke R (2001). Adiponectin gene expression is inhibited by *β*-adrenergic stimulation via protein kinase A in 3T3-L1 adipocytes. *FEBS Letters*.

[26] McMurray RG, Forsythe WA, Mar MH, Hardy CJ (1987). Exercise intensity-related responses of *β*-endorphin and catecholamines. *Medicine and Science in Sports and Exercise*.

